# Working out the manner and cause of death using medicine, marks and micro traces - Case report

**DOI:** 10.1007/s00414-025-03615-x

**Published:** 2025-10-01

**Authors:** Matthias Weber, Pia Rosendahl, Sonja Siegel

**Affiliations:** 1 Landeskriminalamt Nordrhein-Westfalen (LKA NRW) - Institute of Forensic Science, Düsseldorf, Germany; 2 Universitätsklinikum Düsseldorf, Institute of Forensic Medicine, Düsseldorf, Germany

**Keywords:** Blunt force trauma, Marks, Micro trace, Paint, Fracture

## Abstract

This case report describes a homicide in which the victim sustained fatal blunt force trauma to the head caused by an initially unidentified weapon. In addition to soft tissue injuries to the scalp, the victim also suffered a single depressed fracture on the central calvaria located at the os frontale. During the forensic medical examination, the cause of death was determined to be a combination of exsanguination and craniocerebral trauma. However, it was not possible to clearly identify the weapon used based on the soft tissue and bone injuries. Histological analysis revealed foreign material containing iron within the wounds. In the marks examination, the cracks in the fracture of the external plate of the calvaria were compared with potential tools of the crime. Striking similarities were identified regarding the shape and size of the cracks in the bone and scratch marks in the paint coating of a weight plate of a dumbbell that was recovered at the crime scene. Further material analysis confirmed the presence of black, polyester-based coating particles within the bone fractures, chemically identical to the paint coating of the weight plate. These findings led to the identification of the weight plate as the instrument of injury, a conclusion upheld by the court. This case underscores the critical importance of interdisciplinary collaboration within forensic science. Particularly in this case involving blunt force trauma. By integrating forensic medical and histological examination, micro trace analysis and comparative marks examination, the weight plate could successfully be identified as the weapon used.

## Working out the manner and cause of death using medicine, marks and micro traces - Case Report

The examination of injuries resulting from offender-induced force using tools or weapons is typically carried out through a multidisciplinary forensic approach. In addition to the forensic medical assessment of injuries and histological examination of the affected tissues, analyses may include the detection of material residues in soft tissue and bone, as well as tool mark examinations focusing on trauma-induced marks in bone and cartilage tissue. However, in cases involving blunt force trauma, fracture patterns of the affected bones are systematically examined to determine whether the injuries occurred ante-, peri-, or postmortem, and to assess their potential relevance to the cause of death. The applicability of tool mark analysis is often limited to determining the type or class of weapon or object involved [1–5], and only rarely allows for the identification of a specific tool or object as the source of the marks [3, 6]. When the impact surface exceeds approximately 4 × 4 cm, characteristic fracture patterns are unlikely to occur [7].

## Case events

A 58-year-old victim was found dead in the living room of his apartment by his concerned sister, who had authorized access via a key. The body displayed severe head injuries that did not correspond to an accidental fall. The emergency physician on site pronounced death and documented the manner of death as undetermined.

**Fig. 1:**
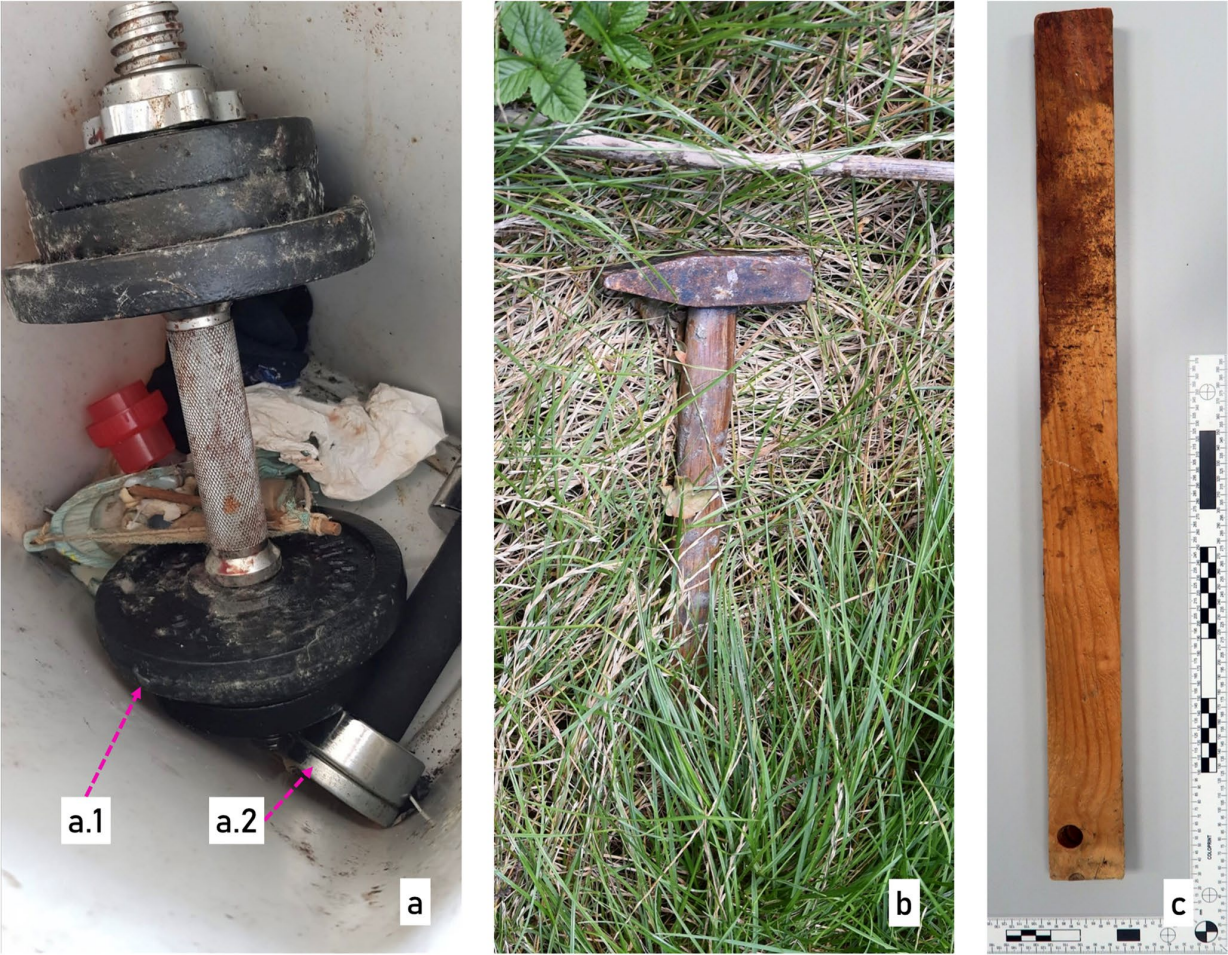
Possible evidence at the beginning of the investigation. (**a**) two dumbbells a.1) dumbbell with black painted weight plates and blood stains, a.2 dumbbell with black handle and silver-colored weights, (**b**) locksmith’s hammer with black coating on hammerhead and wooden handle and (**c**) wooden slat with bloodstains

Further investigations and the results of the autopsy reinforced the suspicion that this was a homicide. During the subsequent crime scene investigation, four potential weapons were secured: a small dumbbell, a large 8 kg dumbbell, a locksmith’s hammer, and a 58 cm long wooden slat (Fig. 1).

The suspect and later accused was very quickly identified as a forty-one-year-old recidivist known to be violent. The defendant met the victim on the day of his release from a two-year prison sentence. When the victim learned of the defendant’s homelessness, he offered to stay with him for a few days in his one-bedroom apartment, where the crime was committed ten days later.

## Examinations and findings

### Forensic morphology

**Fig. 2:**
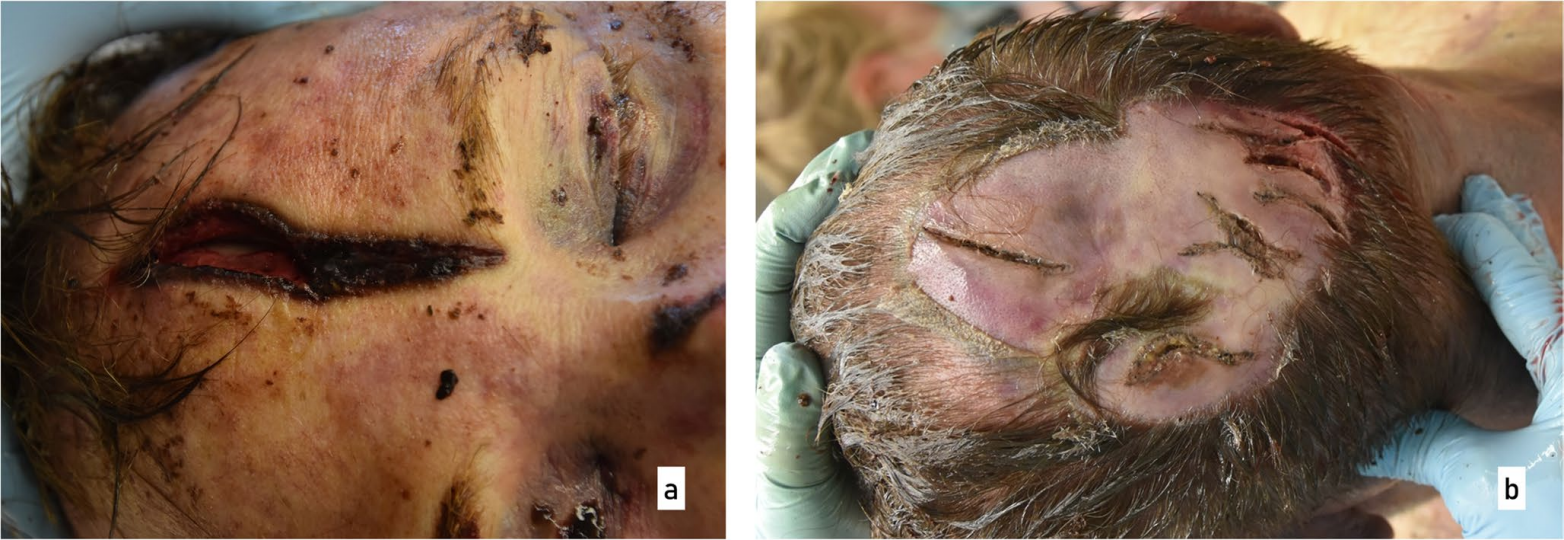
External visual inspection during the autopsy. Injuries caused by blunt force are shown here as an example on the (**a**) victim’s forehead and (**b**) the back of the head

**Fig. 3:**
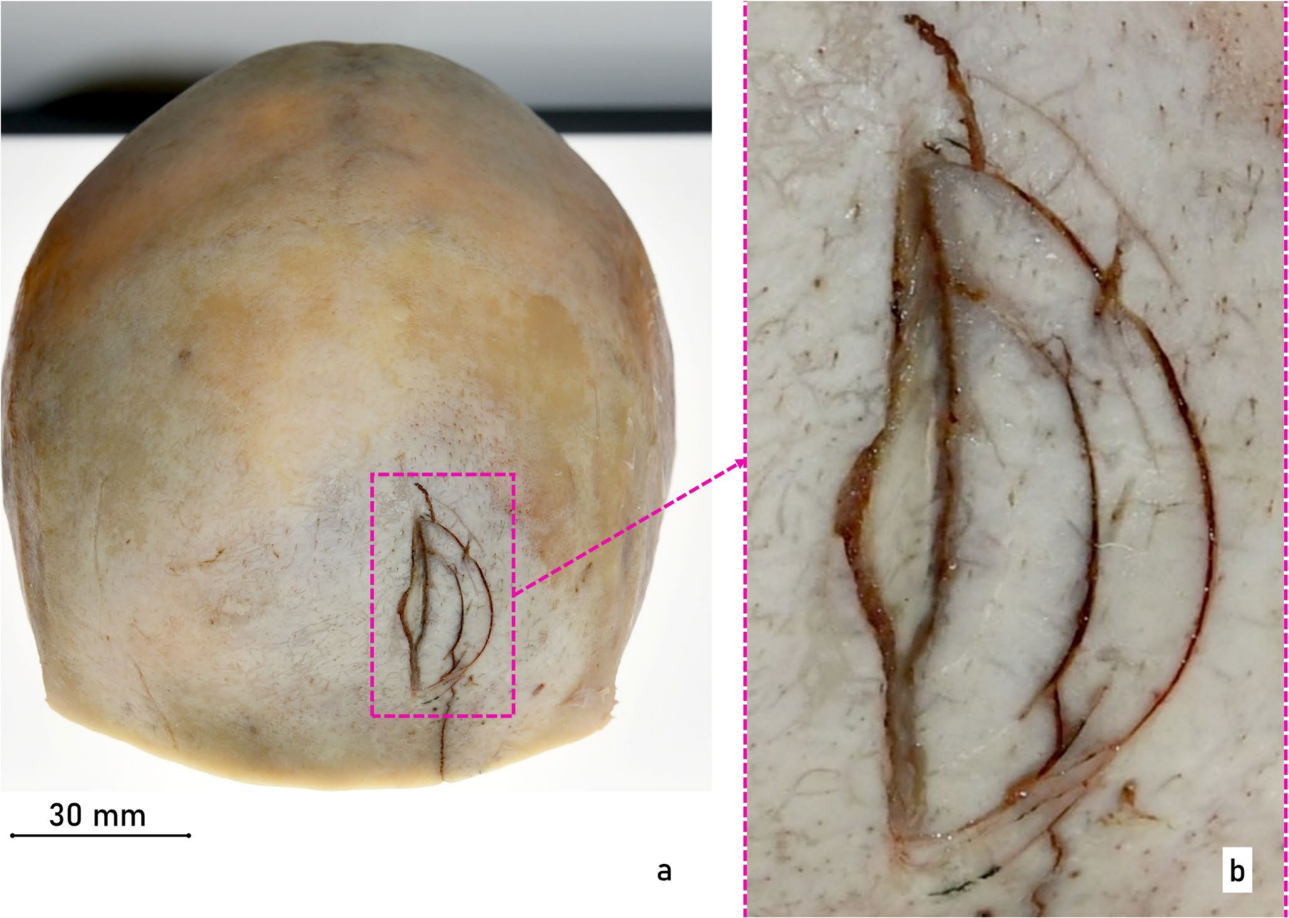
Macerated cranial vault (calvarium) (**a**) semicircular fractures of the left frontal bone (overview) and (**b**) detailed, microscopic image of the fractured area

Prior to the autopsy, a CT scan was performed. The main findings included midface fractures and a fracture of the frontal bone. During the autopsy, numerous injuries to the victim’s head were observed (Fig. 2). In the area of the back of the head, right ear, upper lip, chin and forehead, several lacerations were found resulting in tearing and splitting of the tissue. The most prominent of these was a laceration on the forehead, which appeared as a wide gaping injury with damage to the underlying skull bone. Examination of the skull bone revealed a single depressed fracture located approximately centrally on the frontal bone (os frontale). For further analysis, the calvarium was macerated in warm water without additives. The fracture exhibited relatively straight edges with adjacent, approximately concentric, semi-circular and relatively fine cracks (Fig. 3). Based on the injuries to the soft tissue and bone, however, it was not possible to identify the weapon used, partly because of tissue softening due to early putrefaction changes. Subsequently, the bone was transferred to the police forensic institute for additional examinations of tool marks and micro traces.

In addition to the forensic autopsy, neuropathological examinations were conducted. Despite the presence of mild bleeding under the hard and soft meninges and moderate cerebral edema, the traumatic brain injury was not the sole cause of death. Considering the scalp injuries and the pale tissues, a combination of blood loss and traumatic brain injury was determined as the cause of death. Because of bloodstaining in different rooms at the crime scene, the question arose as to if the victim was able to act after getting hurt. This seemed possible given that the craniocerebral trauma was moderate.

The body was found in the bedroom. However, analysis of the traces of blood revealed that the crime had primarily taken place in the kitchen. It was also established that a considerable number of the blows were inflicted on the victim while he was already in a prone position. The DNA examination revealed cell material (mainly blood) of the victim on the wooden slat and on the heavy dumbbell with black painted weight plates.

### Fine tissue examination

**Fig. 4:**
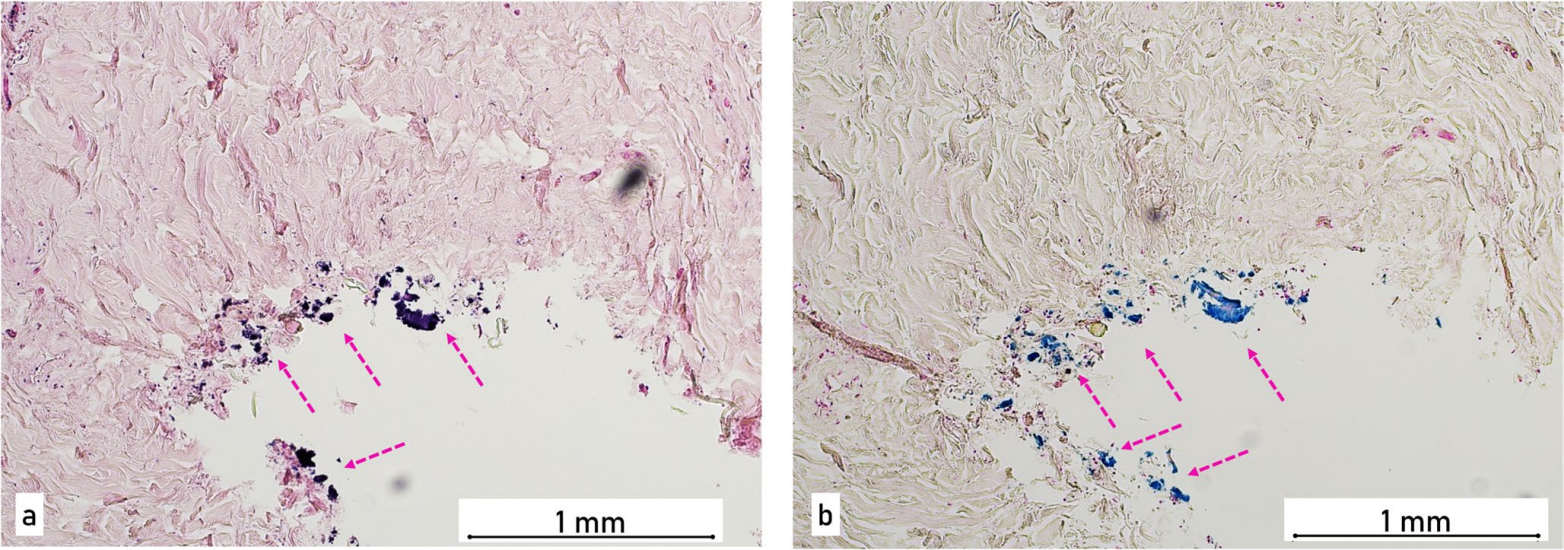
Histological images of the wound edges of one of the injuries on the back of the head 10X magnification (**a**) staining with Hematoxylin and Eosin (H&E), (**b**) iron staining with Prussian Blue

Exemplary samples were taken from the skin and soft tissue of the head injuries for histological examination, in particular to estimate the age of the wounds. After being cut, the samples were embedded in paraffin and stained with haematoxylin-eosin and prussian blue. In some samples, including the wound on the forehead and four injuries at the back of the head, ferrous foreign material was detectable in the tissue of the wound edge. (Fig. 4). In addition, accumulations of erythrocytes and, in some cases, large numbers of granulocytes indicated that the bleedings were not entirely fresh.

### Marks examination

Tool marks are often categorized as impression marks or striation marks. If the tool is pressed statically into the surface of the exhibit and deforms it plastically, an impression mark is created, i.e., a negative pattern of the active surface of the tool. If the tool is moved over the surface of an exhibit of lower hardness by a relative movement and with force, material is removed or displaced and striation marks are created. Since bone is classified as a brittle material and only allows very limited plastic deformation, detailed impressions such as those found in plastic materials are usually not recognizable on bone after blunt force trauma. Rather, the geometry of the impacting surface appears as an impression or perforating fracture allowing the examiner to assess the shape, size, and potentially even the specific characteristics of the causative object [8]. Striation marks in bone can be caused by contact to the bone with sharp blades or by sawing.

**Fig. 5:**
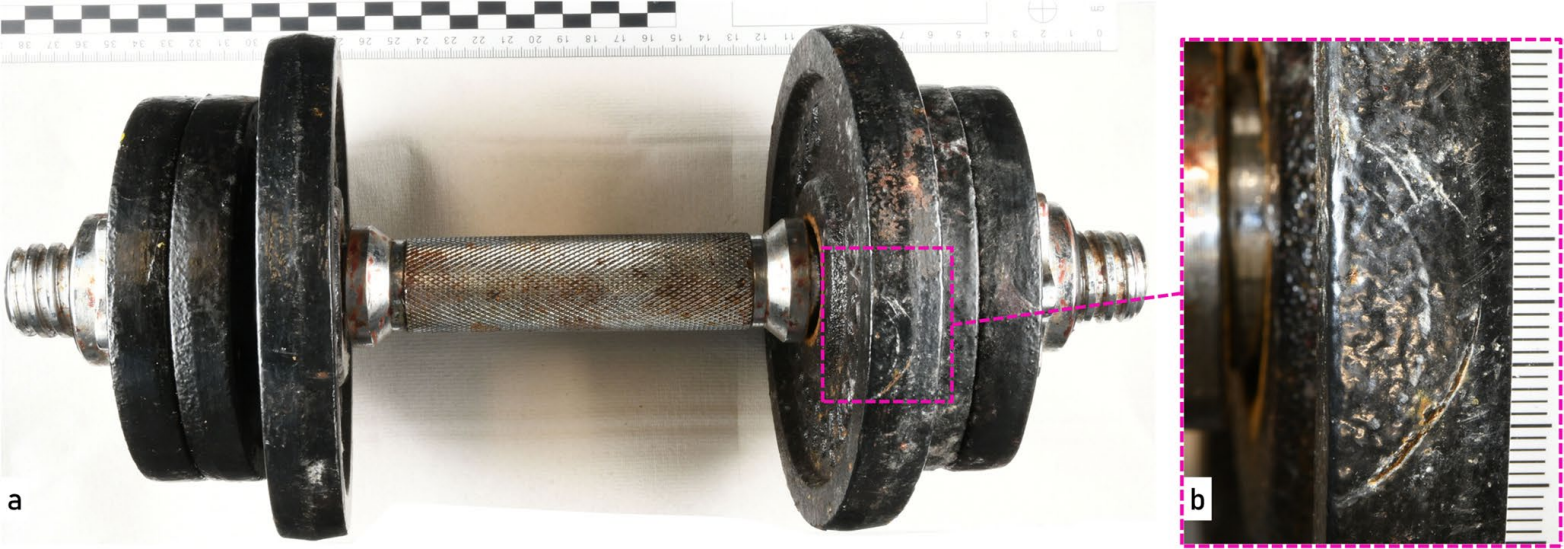
(**a**) overview of the dumbbell with two 2 kg and four 1 kg weight plates and (**b**) detailed view of the scratch marks in the black coating of one of the 2 kg weight plates

No typical tool marks were detected and only the previously mentioned fine cracks of the impression fracture were recognizable. In addition, black material residue was clearly visible in the cracks of the fracture. The next step was to visually examine the tools in question. The only black painted objects in this investigation were the weight plates of the dumbbell shown in Fig. 1-a.1 and the hammer head shown in Fig. 1-b. As the hammer was considered an unlikely cause of the skull fracture in the first assessment due to its geometry and dimensions, the examination of the marks focused on the weight plates of the dumbbell. The dumbbell consists of a silver-colored handle bar, two silver-colored nuts and six black painted weight plates (4 plates labeled “1 kg”, 2 plates labeled “2 kg”). The dumbbell showed large areas of bloodstains and was partially covered in mold fungus. In addition, a mark was found on one of the 2 kg weight plates in the form of superficial scratches in the black paint. The shape of the scratches can be described as approximately concentric and semi-circular and therefore like the cracks found on the fractured skull bone (Fig. 5).

**Fig. 6:**
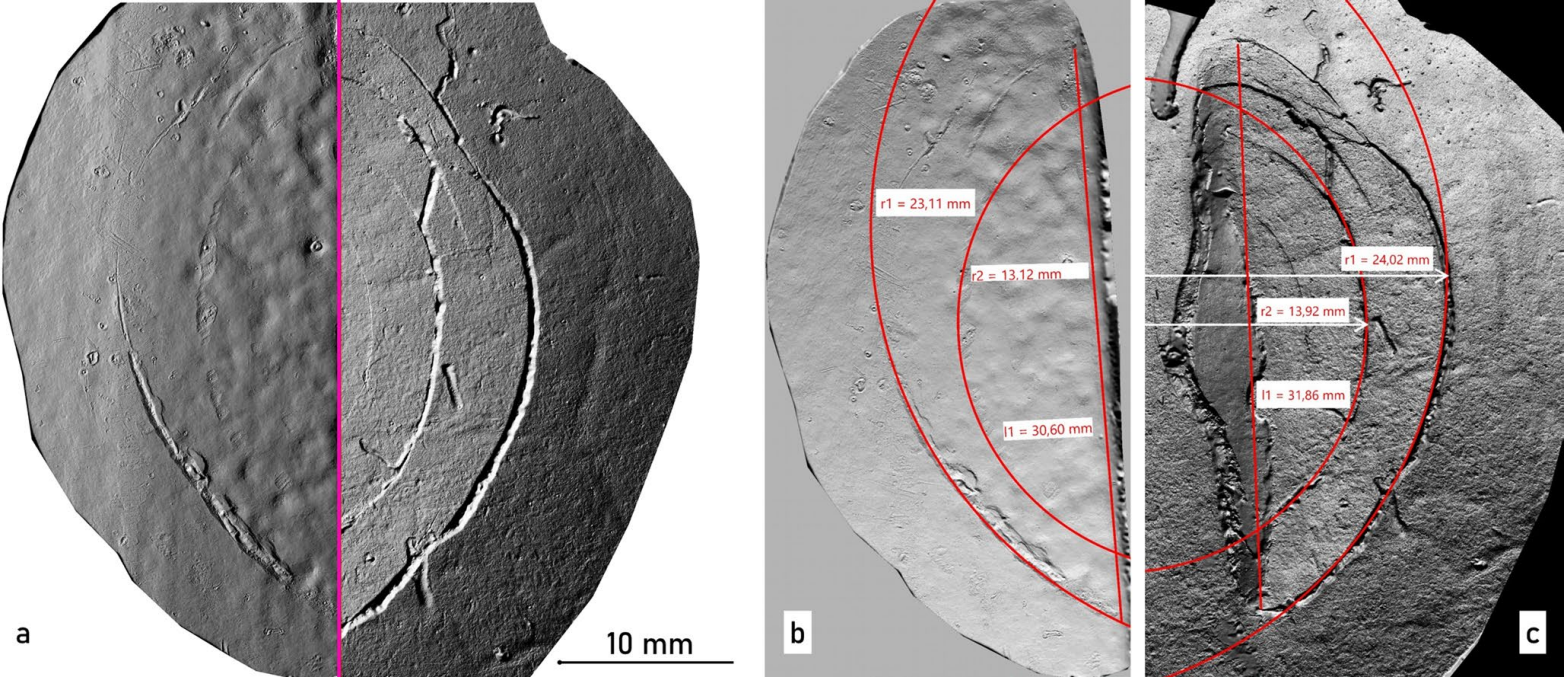
3D-scanned silicone casts of the surfaces of bone and dumbbell. (**a**) Comparative view: scratch marks on the weight plate (left) and cracks within the fracture of the skull bone (right). (**b**) Measurement of scratch marks on the surface of the weight plate. (**c**) Measurement of cracks in the cranial vault

Casts of the scratch marks on the 2 kg weight plate and of the fracture cracks on the skull bone were then made using silicone compound (AccuTrans AB brown, Colténe Whaledent). The casts were scanned using a 3D surface scanner (ToolScan, LIM) and the subsequent comparative examination revealed similarities in shape, orientation, and size between the scratch marks on the dumbbell weight plate and the fracture cracks found in the skull bone (Fig. 6).

The matching morphologies of scratches in the paint and cracks in the bone seem to be mutually dependent. The sequence of the individual events that led to the traces found is likely to have been as follows: When the weight plate hit the head, the rind of the head burst open as a crush wound and the bone was exposed. The bone was fractured by the forceful impact of the weight plate, which was harder in relation to the bone, and the typical crack structures of blunt force, i.e., radial, and concentric cracks, were caused by the compression of the outer bone plate. The sharp-edged cracks in the bone tissue, which is harder in relation to the paint layer of the weight plate, had scratched paint from the surface of the weight plate and left the mark on the weight plate.

Fracture structures are characterized by irregular, individualized patterns resulting from a sequence of directional changes in the fracture, influenced by numerous non-reproducible factors. In the forensic physical fit examination, this is used to prove the former unity of two or more fragments [9, 10].

In the present case, the result of the mark examination was that the similarities found between the crack structures in the skull bone and the shape of the scratch marks in the paint coating on one of the 2 kg weight plates indicate that both surface alterations (weight plate, bone) were caused by the violent impact of the dumbbell on the skull bone.

### Micro trace examination

**Fig. 7:**
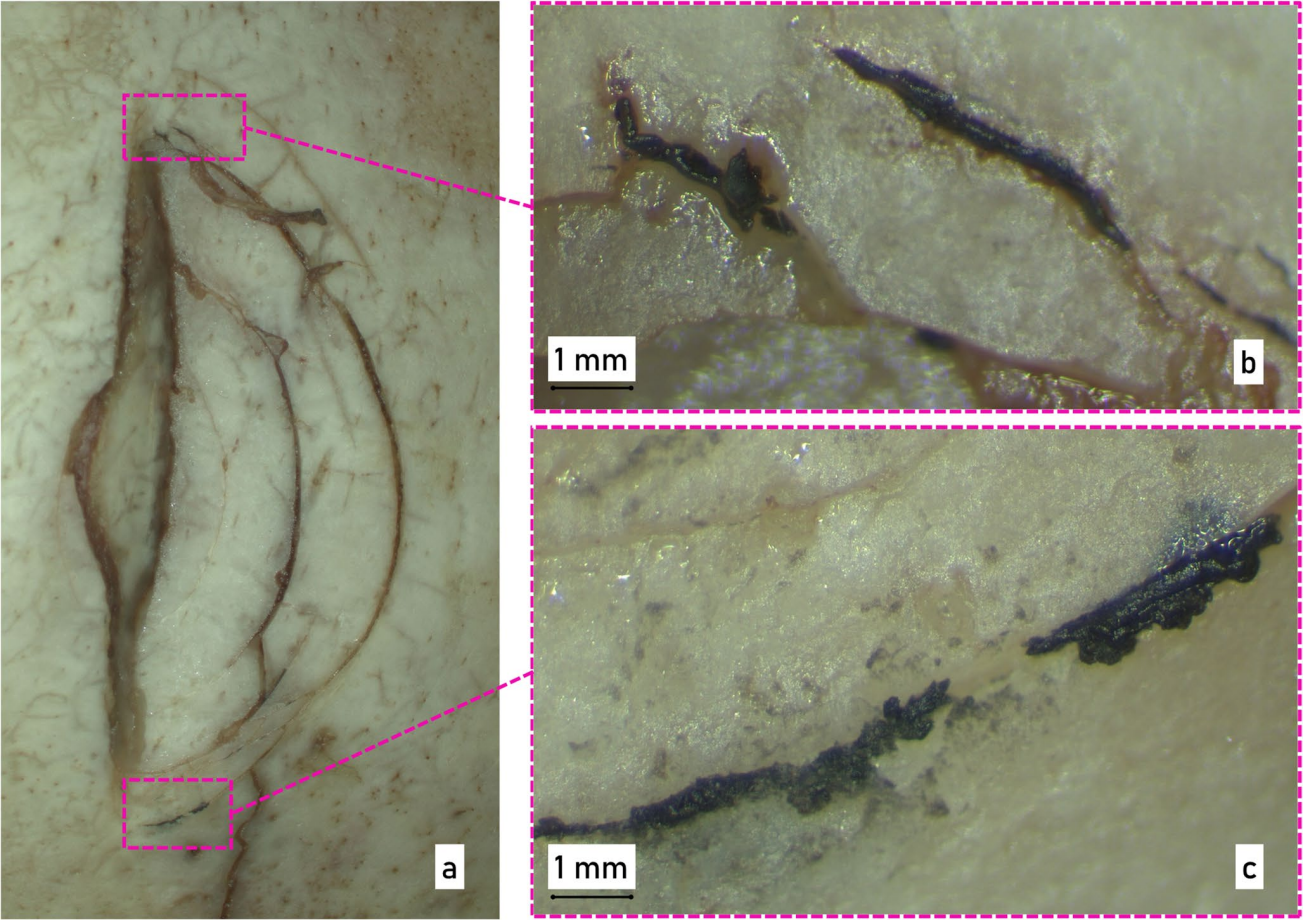
Microscopic Images of (**a**) the surface cracks in the bone, (**b**) and (**c**) detailed images of the material traces found in the cracks

At the beginning of the micro trace investigation, the macerated bone was carefully examined under a reflected light, stereo microscope (magnification up to 100x). The examination revealed conspicuous black material adhering to the outer bone plate (Fig. 7). The particles appeared to have been rubbed into the cracks and are therefore likely to have been caused by the object used in the crime.

The particles in the skull bones were carefully removed from the bone structure using tweezers. Other tools, such as scalpels and dissecting needles, can also be used to remove such foreign bodies. In forensic science, in addition to spectroscopic methods such as Fourier-transform-infrared (FT-IR)- and Raman-spectroscopy to determine binding agents and pigments, elemental analytical examination methods such as scanning electron microscopy coupled with energy dispersive X-ray spectroscopy (SEM-EDX) are also used [11]. To avoid metal contamination, the use of iron-free or steel-free tools based on plastic, titanium or diamond is recommended here. In addition, the utensils are cleaned with wipes and examined under a stereo microscope for foreign matter and the material traces are sampled under the stereo microscope.

The two black-coated potential tools used in the crime, the hammer (Fig. 1b) and the scratched 2 kg weight plate (Fig. 1a1), were sampled and compared with the material samples from the bone using an FT-IR spectrometer (Thermo Fisher, Scientific iN10, infrared microscope, using an IR-diamond cell for preparing a thin, flat sample and measuring in transmission mode). In addition, an elemental analysis of the micro traces from the bone and the weight plate was carried out using SEM-EDX.

**Fig. 8:**
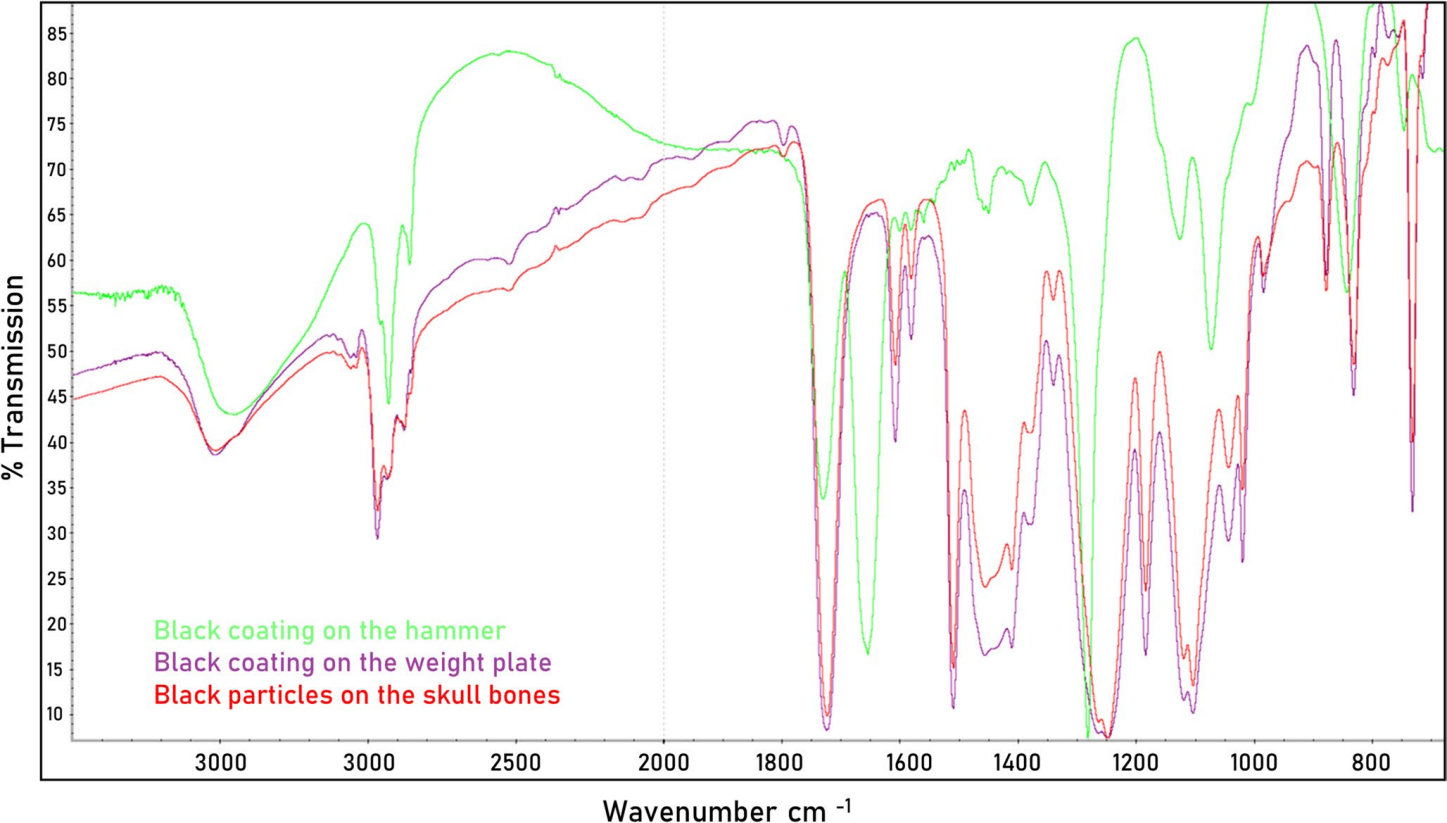
FT-IR spectra matching of the samples from the weight plate (purple spectra), the hammer (green spectra) and from the bone (red spectra)

Due to the non-identical FT-IR curves, the black coating of the hammer could be excluded as the source of the black material traces in the victim’s skull. The FT-IR spectra as well as the elemental composition of the black material traces on the skull and the material of the dumbbell coating (Fig. 8) were indistinguishable from each other and corresponded to a polyester-based coating paint. In general, black polyester-based paints are widely used in the field of coating materials. The micro trace analysis showed that the material in the cracks in the bone was indistinguishable from the paint coating on the 2 kg weight plate, providing moderate support for the hypothesis that the black coating particles originated from the dumbbell and not from another object. However, the material traces do not permit a conclusive identification of the dumbbell as the specific weapon used, as an indeterminate number of objects may share the same type of coating. Nevertheless, the findings represent a significant indication and, in conjunction with the results of other applied forensic disciplines, carry considerable evidentiary value.

The evaluation was carried out in accordance with the ENFSI guideline for evaluative reporting in forensic sciences [12].

## Conclusions and discussion

After blunt force trauma to the head, microtraces such as metallic particles or paint residue often remain on the skull bone [13]. Microtraces can also remain on the tissue after violence such as stabs, chops or cuts and marks left on the tissue when dismembering a corpse. These traces can be analyzed and compared with potential murder weapons and thus make a valuable contribution to the investigation of homicides. In addition to micro trace analysis, the examination of tool marks on human tissue and bone can also provide probative results that support homicide investigations. Sharp force often leads to analyzable tool marks on bone and cartilage tissue [14–19]. For example, chop marks on the skull bone or stab marks on the rib cartilage might result in tool marks that can be used to clearly identify the tool used to commit the crime. However, blunt force trauma to bones rarely produces analyzable tool marks. Fractures in bone tissue caused by tools with an impact surface larger than 4 cm x 4 cm [7, 20] usually result in irregular fracture patterns that provide very limited information about the type of tool used. Tools with a smaller impact surface can produce perforation fractures and depressed fractures, which may at least reveal class and subclass characteristics, such as the size and geometry of the impact surface of the tool. However, identification of the specific tool based on such marks is generally not possible. This case is particularly noteworthy because morphologically distinct tool marks were observed despite the use of blunt force—an uncommon finding in forensic practice. These marks enabled an interpretation regarding the possible causative instrument. Moreover, a cross-transfer of marks occurred—a phenomenon that, to our knowledge, has not previously been described in the forensic literature.

This case vividly demonstrates the high evidentiary power that can be achieved by fully utilizing forensic science in homicide investigations. In this instance, the integration of findings from the forensic disciplines of forensic medicine, material analysis, and tool mark examination leads to the identification of the weapon used and at least a partial reconstruction of the events during the commission of the offense. The results of the forensic medical examination revealed that the crime must have involved blunt force against the victim’s head and a combination of blood loss and traumatic brain injury as cause of death. The histological examination found iron residues in the skin wounds. The marks examinations revealed corresponding scratch marks on one of the weight plates of the 8 kg dumbbell and the cracks in the skull bone. The micro trace analysis showed that the material in the cracks was indistinguishable to the paint coating on the dumbbell plate.

In addition to these results, the blood stain pattern analysis and the DNA analysis contributed to further important findings that helped to elucidate the case. The blood stain pattern analysis showed that the crime must have taken place in the kitchen and that the victim was partially lying down during the crime and was therefore defenseless. The DNA analysis showed the victim’s blood on the 8 kg dumbbell and the wooden slat.

Based on the evaluation of expert reports on forensic medicine, DNA analysis, micro trace analysis, and tool mark analysis, the court considers the dumbbell to be the murder weapon. This demonstrates the generally high value of micro trace analysis in cases of homicide involving blunt force trauma and further emphasized that even in such instances, the analysis of the tool marks on the bone can yield valuable results. Therefore, the authors advocate that both analyses should be standard procedures in forensic investigations of bone and cartilage tissue in sharp and blunt force trauma, as well as in cases involving dismemberment.

The court thoroughly examined all forensic expert opinions and evaluated the circumstances of the crime as follows: The defendant struck the victim multiple times on the head with the 8 kg dumbbell in the kitchen, resulting in fatal injuries. Consequently, the defendant was convicted of manslaughter and sentenced to 10 years in prison.

## References

[1] Ta’ala SC, Berg GE, Haden K (2006) Blunt force cranial trauma in the Cambodian killing fields. J Forensic Sci 51(5):996–1001 17018075 10.1111/j.1556-4029.2006.00219.x

[2] Clark EG, Sperry KL (1992) Distinctive blunt force injuries caused by a crescent wrench. J Forensic Sci 37(4):1172–1178 1506833

[3] Zugibe FT, Costello JT (1986) Identification of the murder weapon by intricate patterned injury measurements. J Forensic Sci 31(2):773–777 3711845

[4] Otero F, Béguelin M (2019) Experimental Study of Cranial Injuries Due to Blunt Force Trauma: Sus Scrofra Domestica Model. J Forensic Sci & Criminal Inves 13(555856)

[5] Mittal P, Bohnert M (2022) Firearm as a blunt weapon – three cases of pistol whipping and a review of the literature. Forensic Sci Int Rep 6:100299

[6] Zugibe FT, Costello J, Breithaupt M (1996) Identification of a killer by a definitive sneaker pattern and his beating instruments by their distinctive patterns. J Forensic Sci 41(2):310–313 8871391

[7] Geserick G (2003) *Verletzungen des Knöchernen Schädels (Hirn- und Gesichtsschädel)*, in *Handbuch Gerichtliche Medizin*. Springer, Berlin Heidelberg, B. Brinkmann and B. Madea, Editors

[8] Ramsthaler F et al (2019) Hammer blows to the head. Forensic Sci Int 301:358–370 31212143 10.1016/j.forsciint.2019.05.045

[9] Weber M, Rothschild MA (2020) Passspurenuntersuchungen – Fallbeispiele Zur untersuchung Aus dem Körper geschädigter präparierter fragmente Nach stumpfer und scharfer Gewalt. Rechtsmedizin 30(3):168–174

[10] Prusinowski M, Brooks E, Trejos T (2020) Development and validation of a systematic approach for the quantitative assessment of the quality of duct tape physical fits. Forensic Sci Int 307:110103 31874301 10.1016/j.forsciint.2019.110103

[11] ENFSI (2022) *Best Practice Manual for the Forensic Examination of Paint*; Version 002 https://enfsi.eu/wp-content/uploads/2022/11/EPG-BPM-001.pdf

[12] ENFSI (2016) *ENFSI guideline for evaluative reporting in forensic science (*https://enfsi.eu/wp-content/uploads/2016/09/m1_guideline.pdf). Version 3.0

[13] Vermeij EJ et al (2012) Analysis of microtraces in invasive traumas using SEM/EDS. Forensic Sci Int 214(1):96–104 21871744 10.1016/j.forsciint.2011.07.025

[14] Bonte W (1975) Tool marks in bones and cartilage. J Forensic Sci 20(2):315–325 1123601

[15] Clow C (2005) Cartilage stabbing with consecutively manufactured knives: a response to Ramirez v. State of Florida. AFTE J 37(2):86–116

[16] Froch-Cortis J, Skarupke B, Weber M, Rothschild MA (2016) Silikonabformungen am Knorpel Als Werkzeugspurenkundlicher „fingerabdruck. Rechtsmedizin 26(3):169–176

[17] Locke RL (2008) Application of the dynamics of a knife puncture to identify toolmarks in a cervical vertebra. AFTE J 40(2):137–142

[18] Weber M, Cortis J, Rothschild MA (2015) Toolmarks in human cartilage and bone - ten case studies. AFTE J 47(2):79–86

[19] Weber M, Niehoff A, Rothschild MA (2021) Insights to enhance the examination of tool marks in human cartilage. Int J Legal Med 135(5):2117–2134 33987743 10.1007/s00414-021-02609-9 PMC8354928

[20] Holz F et al (2018) Frakturen des Gehirnschädels Als folge stumpfer Gewalt. Rechtsmedizin 28(3):229–240

